# An Address Read before the Esculapian Society of the Wabash Valley

**Published:** 1858-01

**Authors:** E. Read

**Affiliations:** Terre Haute, Indiana


CHICAGO MEDICAL JOURNAL.

VOL. I. JANUARY, 1858. NO. 1.

### ORIGINAL COMMUNICATIONS.

### ARTICLE I.

### AN ADDRESS READ BEFORE THE ESCULAPIAN SOCIETY OF THE WABASH VALLEY,

HELD AT PARIS, ILLINOIS, ON THE 29TH OF OCTOBER, 1857.

BY E. READ, M. D., OF TERRE HAUTE, INDIANA.

MR. PRESIDENT AND GENTLEMEN OF THE ESCULAPIAN SOCIETY:

I have witnessed with the profoundest pleasure the proceedings of your Society, from its formation up to the present time, and although I have not had the good fortune to meet with you at any time before this, yet I have been apprised of the action of each one of your meetings, which, I can assure you, has received my heartiest approbation. I am gratified to have the privilege of meeting with you on this occasion, and I am still more gratified, to witness so much earnestness on the part of intelligent medical gentlemen in the pursuit of knowledge, and in the desire of elevating, if possible, the most dignified, the most useful, and the most humane of all professions.

I regret, however, my inability to aid you in your laudable undertaking, as much as I could wish, or to bring you an offering, which would make you wiser or better physicians.

So far as I can, however, in the short time I have appropriated to this purpose, I will offer for your consideration some of the changes I have witnessed in the treatment of diseases since I commenced the practice of medicine. I may also advance some theories, which may not be consonant with your own views, but, if by so doing, I may direct your attention to doctrines, which, in my opinion, should long since have been expunged, notwithstanding you may disagree with me, yet I shall flatter myself that you will unite with me in investigating the subjects I shall present, and in earnestly seeking for what may be true and what false. And I may here be indulged in the expression of opinion, that there is nothing so hurtful to advancement in science and knowledge as prejudice, which consists, in the sense I here use it, of opinions so long taught, that they are regarded too sacred to be changed. It is a part of our nature to venerate the things which have been taught us, as venerated by our fathers before us. It is one of the great struggles of life, to abandon those things which we have loved and cherished.

It is the blotting out of impressions, which have been the dream-like ideals of all that is beautiful and good. It is striking out the sunlight of our memories, and giving to us the whirlwind and the storm. It is inviting us from that which we have been taught is true and reliable, to that which is unknown and unexplored.

Is it, then, not wonderful that any should have the temerity to abandon the known for the unknown?—that any should be found willing to sacrifice the luxurious ease already prepared for enjoyment, for the toils and anxieties incident to the investigation of subjects not well settled? Hence, we find, that doctrines once established and once received, are difficult to change. Few are found willing to attack them — few bold enough to lay the axe at the root. In the primitive ages, and in the primitive state of our art, it was even more difficult than now, because all knowledge was founded on observation, and any deviation from that was a criminal offence.

The sick were in early ages exposed in public places, so that any who passed by, and who had been similarly attacked and cured, might give their advice for the benefit of the sufferers. At a somewhat later period, another method was adopted, which was an improvement, and which was better calculated to accelerate the progress of the art, and at the same time more humane, because the sick were not exposed to the public gaze.

It was this: all who were cured of disease were required to go and make an inscription in the temples of the symptoms of their disease and the curative agents which had been beneficial to them. These records were kept with the same care as the archives of the nation. They were religiously preserved for the benefit of the diseased, and for a long time every one had access to them, and had the privilege of choosing for his own sickness or that of his neighbor's the mendicaments of which experience had taught and confirmed the value. This was, perhaps, the very best and safest method which could at that time have been adopted. It was the knowledge exclusively of observation which has ever been recognized as of so much value. From this rich depository of facts, written with simplicity and truth, correct and reliable principles were deduced for the practice of medicine.

But presently, as these were all written in that temple, the many were excluded and the priests had the entire control and became by law the privileged class, having the exclusive right to practise medicine. Out of this great roll of facts they formed a medical code, a kind of digest, which was called the *sacred book*, and from its directions they were never permitted to vary.

How many at this day, let me ask, have their sacred books of practice, from whose precepts, right or wrong, they never vary? If, in following the rules there laid down, they could not save their patients, they were not held responsible, but on the contrary, they were punished with death if, after departing from them, the result did not justify their course.

It is not presumed that many would dare transcend a law so rigid and exacting, hence all progress was arrested, and the sacred pages alone were regarded incapable of having faults upon their face. The correctness of their principles having been first established they became the law. It was regarded that practice confirmed by long experience and supported by the authority of the greatest masters of the art, was preferable to the limited experience of each particular physician.

It is a singular coincidence, and shows either a common origin of the races or the justness and universality of the law, that at a period nearly four thousand years distant, and in our own continent, and among its wild savages, we find a very similar usage to prevail.

I shall scarcely deem an apology necessary for using the language of Surgeon Moses of the U. S. army, in describing the usages of the Chinook Indians, who reside in Washington Territory, in regard to the treatment of the sick by their medicine men.

In ordinary cases of sickness, the aid of the medicine man or doctor is called in. This individual is held in high estimation, and demands large fees for his advice and services; these are given at a vast personal risk, and somewhat upon the terms of their advertising professional brethren in large cities.

Upon visiting the patient and receiving his fee, the doctor goes actively to work to drive out the evil spirit from the suffering body, where it has assumed the form of a wolf, a beaver, or large stone.

Should the unhappy victim of Esculapian art fortunately get well, the doctor remains in the enjoyment of his professional gains. Should death, however, have knocked at the door of the lodge during these mockeries, as he invariably does in some cases, the doctor not only has expended his time and labor for nothing, but now has forfeited his life, by failing to restore his patient to health. If he can compromise the matter with the relations and friends of the deceased, by paying his value, estimated in horses, blankets, canoes, he redeems his own life, but failing to satisfy the demands of the afflicted, who are very exacting, he may not expect to live to see the sun rise many times.

In this case, no less than in that of the priests of Egypt, who were governed by the *sacred book*, there was but little temptation to depart from known usages and precepts. I am just here reminded of the propriety of correcting a very popular error in regard to Indian knowledge of the medicinal virtues of the plants of the country and of their wonderful skill in curing diseases.

I have examined with much care, the reports of our army surgeons, who have been stationed in the Indian country at the north, east, south, and west. Their universal testimony is, that they have no knowledge of diseases and have none of the medicinal virtue of remedial agents. They declare them incapable of curing a snake bite, or any of the simplest diseases which common experience, with common foresight, would seem to teach. Their females suffer greatly from uterine diseases, and have as much parturient suffering, as falls to the lot of those in the enjoyment of the luxuries of civilization. We so frequently hear of "Indian doctors," who can cure all diseases, because they have derived from the Indian, directly or indirectly, this treasured wisdom, that it is well to keep ourselves advised of the truth, that we may counteract and crush out this kind of humbuggery.

A few years ago I acquired a good deal of reputation with a band of Ottoe Indians by an antimonial prescription, which was so happily suited to their cases, that they insisted on having as much of the medicine to last their journey, in case they needed it. They had a medicine man with them, but he made no pretensions to skill, and was amazed at the prompt and efficient action of the medicine I administered.

When I commenced the practice of medicine, there was no remedy in such universal repute as bleeding. It was regarded absolutely necessary to premise any treatment by letting blood. No other remedy could be used as a substitute. No matter what the disease, the amount of the circulating fluid must be diminished. If any by chance escaped, it was then regarded doubtful whether recovery were possible. An almost universal mania had seized upon the minds of physicians, and teachers and writers recommended it as a panacea. To be an expert phlebotomist was almost as valuable as to be the prince of surgeons, and one who had a reputation of this kind could see the blood flow from morning until evening without leaving his office, from the army of droppers in, to be bled, because they were not exactly well, or had some real or imaginary disease.

The women had to be bled when they were pregnant, and when they were not, with a view of becoming so. The girls had to be bled for amenorrhœa, and when spring came there was a universal bleeding and drinking of sassafras tea. I became so accustomed to the appearance of the blood at that time, that from it alone I could form a pretty good idea of the disease without any other aid. I was for a short time connected professionally in Cincinnati with a very excellent physician, a graduate of the University of Pennsylvania, and I saw him bleed a man for dropsy for twenty consecutive days. There was scarcely any coloring matter left in the blood, and he was terribly pale and death-like. As long as he dared, he bled him. He recovered, however, from the dropsy, and the physician was pronounced the best dropsy curer in the city. The bleeding was resorted to in that case under the impression that it increased the absorbent system and stimulated it to greater activity, and in that way carried out of the system the excess of water.

This method of treating dropsy was that recommended and used by Hippocrates, who was a great bleeder. He cured enlargement of the spleen and dropsies by venesection. He bled boldly and freely, even to fainting, and would sometimes open veins in both arms at once. He informs us that he bled occasionally in the hands, ankles, hams, forehead, tongue, nose, behind the ears, the breasts, and under the arms. Speaking of the nose brings to my mind some one that wished me to bleed him in the point of the nose many years ago. I suppose some believe Hippocrates recommended it. I found it an easy matter to obtain blood, and repeated it in his case several times. Dr. Rush was a great admirer of Hippocrates, and I presume the bleeding in dropsy came into vogue through him. He speaks of it as the first remedy to be used in this disease. Dr. Monroe quotes a case of dropsy from Epenius, in which bleeding succeeded, but not till after it had been used twenty times. "I could add," says Rush, "the histories of many cases of anasarca and ascites performed by means of blood-letting, not only by myself, but by a number of respectable physicians in the United States. Indeed, I conceive this remedy to be as much indicated by a tense and full pulse, in these forms of dropsy as it is in a pleurisy or in any other common inflammatory disease."

The aged and infirm, the strong and robust of middle life, and the tender little infant, were all alike amenable to the law which declared bleeding to be omnipotent. I have impressed indelibly on my mind one of the first cases I ever had the charge of. I was called just after daylight one fall morning to ride three miles in the country to see an infirm and delicate old lady of 65 years, who was said to be in a singular condition. When I arrived, I saw my patient sitting in a rocking chair, dressed with great care and neatness, but having a wild and frightened look. She was constantly picking at her dress, as though removing something disagreeable to her. She had the exact appearance of one with delirium tremens. She gazed intently upon me, but made no response to my interrogations. I examined her carefully, for she made no resistance, although she seemed alarmed. As you might expect, I found her tongue with a white coat upon it—a very small, feeble and frequent pulse. I was a good deal puzzled. I hesitated in my own mind whether it was inflammation or a nervous disease. It had come on suddenly. She had gone to bed the evening previous in her usual health. At length, I resolved it to be some kind of phrenic and insidious inflammation, that required the lancet. I bled her, applied a blister to the back of her neck, gave her ten or fifteen grains of calomel with a little rhubarb, and in twenty-four hours she was dead. Under any treatment she may have died, but I am persuaded I was myself greatly benefited by the case. It led me to reflect more, and to question the truth of all which is written and taught.

If a little child at that time was seized with any disease, which created excitement in the circulating system, the physician turned its head to one side and opened the jugular vein with the greatest possible adroitness. The venous system was carefully studied with a view to the nicest selection of veins in different parts of the system—the neck, the arms and the feet. The old ladies had a wonderful penchant for foot bleeding.

I used frequently to bleed the medical gentleman referred to in the case of dropsy. His preference was the temporal artery, and I could with skilful nicety cut down upon it and then make my puncture. From constant use, most physicians were skilful phlebotomists or venesecters.

This ancient and almost universal method of treating diseases probably had its origin in the humeral pathology, and this was regarded a very direct method of ridding the system of some of its peccant and morbid humors. It received, however, for a time a check, when the doctrines of Cullen began to be received in the profession.

He taught in opposition to the ancient theories, that the first changes induced in the animal system by the operation of the exciting cause of fever is a diminution of the energy of the brain; that all the powers of the body and all the faculties of the mind, that the functions of sensation and motion, the process of respiration, circulation and secretion, all fail, or are diminished in the general debility; that after a certain time a morbid increase of some of these functions, especially of the circulation, takes place, with an augmentation of the heat; that these three states, that of debility, of cold and of heat, bear to each other the relation of cause and effect; that the first state is the result of the sedative or debilitating influence of contagion, marsh miasma, cold, or any other exciting cause, and the subsequent states the result of the first; that the debility produces all the phenomena of the cold stage, and especially a spasmodic constriction of the extreme arterial vessels; that this spasm or atony of the extreme vessels exists not only in the first attack of the cold stage, but remains during the whole subsequent course of the fever; that the spasm of the extreme vessels throws a load of blood on the central parts of the circulating system, which proves a source of irritation to the heart and arteries, and excites them to a greater action; that this increased action, the source of the heat and the other phenomena which constitute the second or hot stage, continues till the spasm is relaxed or overcome; and that this excitement of spasm for the purpose of producing the subsequent reaction is a part of the operation of the *vis medicatrix naturæ*, the innate preserving power of the constitution.

In his own language, this distinguished theorist says our doctrine of fever is explicitly this: The remote causes are certain sedative powers applied to the nervous system, which, diminishing the energy of the brain, thereby produces a debility in the whole of the functions, and particularly in the action of the extreme vessels. Such, however, is at the same time the nature of the animal economy, that this debility proves an indirect stimulus to the sanguiferous system; whence, by the intervention of the cold stage and spasm connected with it, the action of the heart and large arteries is increased, and continues so till it has had the effect of restoring the energy of the brain, of extending this energy to the extreme vessels, of restoring therefore their action, and thereby especially removing the spasm affecting them; upon the removing of which, the excitation of sweat and other marks of the relaxation of the excretories take place.

Brown, the pupil of Cullen, taught a like doctrine; except that he regarded fevers even more the result of a debilitating agent; that it was the greatest debility compatible with life and not long compatible with it. During the reign of the doctrines of these extraordinary men, blood-letting was not in accordance with their notions, and stimulants and roborants were the remedial agents employed. These were generally esteemed doctrines of heterodoxy, although in reality they were greatly in advance of the age in which they lived. As their novelty wore away, they were cast aside for the more ancient notions, which now had engrafted upon them inflammatory doctrines that called more loudly than ever for the lancet. At the head of this class of medical philosophers in our own country was the celebrated Dr. Rush, who acquired a world-wide reputation for the doctrine which he advanced and practised upon, to bleed in the yellow fever, and to administer his dose of ten grs. calomel and ten of jalap, which acquired the sobriquet of Rush's ten and ten.

A more enlightened era having dawned upon medical science, this disease is now treated upon more rational principles.

The influence which Rush had acquired in America by his writings and public teachings had much to do, I have no doubt, in confirming in the minds of medical men not only the utility but the absolute necessity of blood-letting in most diseases.

Even at this day, our schools teach the propriety of bleeding in pneumonia, and its general antiphlogistic treatment. For many years I followed this treatment, which, however, almost always failed in accomplishing what I expected, and consequently did not meet my approbation. I followed it no longer than I found a more rational and successful method of treating the disease.

I was forced to relinquish my preference for cherished notions when I found that bleeding and antimony did not cure the disease. I was forced to abandon a treatment that so rapidly diminished the powers of life, when I saw its pale and haggard victims with their shattered constitutions, and when I saw those advanced in life so especially obnoxious to it, in whose constitutions the possibility of inflammation could scarcely exist. Does not reason and common sense abjure all antiphlogistic notions in a class of patients like those I have here cited? And I would appeal to the intelligent medical gentlemen who are my auditors, if our pneumonic or winter-fever patients are not of those whose constitutions are broken with previous disease, mostly ague, and those advanced in life. Is not the winter and spring peculiarly the season of death to the old and infirm, and the disease of which they die pneumonia? Would you not rather tranquilize the system and equalize the circulation, and maintain the diminished and wasting powers of life with opiates, stimulants and tonics? I might enter at length upon the pathology of this disease had I leisure. In this region its study is pre-eminently important, as it is the prevailing disease in the winter and spring months.

Last year, I had a little son absent from home at school. I received in the month of February a letter from his teacher, saying that he was seized with a violent disease and was very ill. I immediately took the cars, and the next morning was with him. As I entered the gate I met the attending physician, just leaving from his morning visit, who very politely went back with me to give me the history of the case and his treatment. Four days before that, at night, he was exposed at a public exhibition, in a heated and crowded room, to a draft of cold air. Before morning, he was seized with violent and suffocating coughing, with pain in the side, and the doctor was sent for before daylight. On his first visit, he informed me that he bled him, and applied a blister to his side, and immediately administered a dose of calomel. As soon as that had operated, he put him on antimony, which very soon acted on his bowels, and for two days and nights he had involuntary discharges every few minutes. To check these, he gave small doses of calomel, and when I met him on my arrival, he had just given his morning dose of calomel, and had left antimony to use through the day. His skin was hot and dry, and his pulse very much excited. The doctor said that he regarded it very strange, that his skin did not become moist under so free a use of the antimony, but he intended to give it in increased doses that day to induce perspiration. After the doctor left, I sent to a druggist and procured a little Dover's powder and quinine. I immediately gave him a few grains of the Dov. powder, and in twenty minutes he was bathed in a profuse perspiration, which continued under the repetition of the powder once in six hours. In the meantime I gave him quinine freely, and in a few days he had recovered, except his strength, which required a good while to regain. When the doctor made his evening visit (for I requested a continuance of his calls, although I did not expect to use his medicine or his knowledge), he was amazed to see the favorable change which had taken place, all of which he ascribed to the virtues of his antimony. I did not undeceive him, because I thought he was too old to learn, and would think that I was disposed to find fault with his practice. He was a feeble little boy, not fourteen years of age, and had been subject to just such attacks all of his life, and was always cured by a teaspoonful of paregoric and a little sweet oil. The doctor regarded it pneumonia, when in fact it was spasmodic croup, and he had accordingly resorted to the most heroic antiphlogistic treatment to cut short the terrible inflammation which he thought would speedily consume him.

Now, in this case, any one can see that the child's life was really endangered by the medicines, and that in all human probability he would have died from antimony if the treatment had not been stopped. Whenever, in any disease, involuntary discharges from the bowels are induced from the use of antimony, every additional dose positively *endangers* life. There is no remedy more potent for good or for evil than this, and its use requires the greatest care and caution. I have long since abandoned it in the treatment of pneumonia, and have substituted remedies which I regard more in accordance with its pathological condition, and which in my hands have been greatly more successful than when I relied upon the antiphlogistic treatment.

In the treatment of fevers, when I first became connected with the profession, emetics, purgatives, alteratives, diuretics, and diaphoretics, were all liberally used. Emetics, after bleeding, were resorted to; then whatever morbid matter was left in the system, was to be purged out, according to Hamilton's theory, whose work on the use of purgatives was greatly admired. If, by chance, anything was left after that, the skin and kidneys were required to finish the work. The theory of critical days had not yet been laid aside, and the 7th, 14th, and 21st, were anticipated with fear and trembling. It was not thought that a fever could be cut short from a definite course. Patients were almost starved, and cold water, except in a limited quantity, was carefully withheld. Medicine was given almost hourly, which, with the disease, exhausted the powers of life very rapidly, and a much larger proportion died then than now. They seemed to entertain very curious notions in regard to the secretions, and it was rarely, if ever the case, that the doctor could be satisfied on this point. There was too much or too little bile in the system, and the liver was blamed for everything. The tongue had to be perfectly clean, before tonics could be resorted to; the consequence was that the patient either died or recovered before the term had arrived, when prudence and propriety indicated their use. It is gratifying to reflect, that with the rapid progress, which has in the last few years pervaded all pursuits of life, the science of medicine has kept pace, shedding its blessings upon the whole human race, and sparing from pain and death those, who most of all others, look to us for aid and sympathy.

Epidemics, which formerly terrified the nations by their desolating and unchecked progress, have been almost shorn of their terrors. Vaccination has stayed the prevalence of small pox, and cholera is less fatal than formerly. Human suffering is shortened and human life lengthened.

The hospital reports of Paris show that while in 1805, one in seven of those admitted died, now but one in twelve; thus showing that our science has increased in its ability, in the same buildings, in a little over fifty years, to save life seventy-one per cent. In other words, when formerly fourteen men died in each hundred admitted, now only eight die, a saving of six persons in a hundred. Not only is the mortality in this wise lessened, but the duration of disease is diminished. Formerly, the residence in the same hospitals referred to was thirty-nine days, now twenty-four—a difference of fifteen days since 1805.

In the treatment of special diseases, the improvement has even been greater. In the same time, one in fifty-six cases of syphilis died, now one in two hundred and ninety-four. According to Dupin, in France, the duration of life has been increasing, equal to fifty-two days for each year, from 1776 to 1842, or nine and a half years for the whole period. The increase per annum was no time less than nineteen days.

In London, at the present time, one in forty dies, formerly one in twenty died.

In obstetrical practice, an hundred and fifty years ago, one in forty died, now less than one in two hundred and fifty. The statistics of the hospitals of our own large cities show results similar to those in Paris. In the last seventy-five years, life has been prolonged more than twenty-five per cent, and the duration of treatment lessened more than one-third.

Facts are not to be disputed, and I have referred to these with peculiar pleasure as the highest evidence of the wonderful advancement in the science of medicine. Surgery, too, is disrobed of most of its terrors and pains—a blissful sleep overtakes the subject, from which the keenest blade does not arouse him. Our own country has contributed largely to these advancements. Chloroform was an American discovery. But recently a gentleman of Georgia has discovered a new class of nerves, which he calls the excito-secretory. England and France claimed the discovery, but an investigation gave the palm to our own. Marshall Hall, just before his death, declared it an American discovery. In pathological investigations, I think American physicians are in advance of all others. And in topography, the work of a western physician, Dr. Drake, has no parallel. It is the most learned work that has ever been written on that subject, and I doubt whether any other age can produce a similar one.

Dr. Armstrong, who has written the most learned work upon typhus fever in the English language, and who advocated opinions greatly in advance of the age in which he lived, had much to do, I have no doubt, in introducing a more rational treatment of fever. It was long after his death before his views obtained a favorable reception, but his strong reasoning gradually took hold of the minds of men and led them to think and reflect.

Marshall Hall's work upon the loss of blood was another lever that was brought to upturn many of the old notions, and when we add to that the universal scourge from 1830 to 1833 of all Europe and America by cholera, a disease which clearly indicated a stimulant and tonic treatment to maintain the powers of life which in a few hours were wasted away, we have a combination of causes which wrought a change in the sentiment and practice of medicine.

Another cause I will add, which I have no doubt has had a considerable influence in increasing our confidence in *vis medicatrix naturæ*, which is the doctrine introduced by Hahnemann, and known as that of homœopathy.

We either had to concede that the doctrine of infinitissimal doses had some curative effects, or that nature, left to its own recuperative powers, could restore to health. The latter was most in accordance with our pride of opinion, and was generally adopted; and in adopting it, we were compelled to relax our hold upon the heroic doses which had so generally been adopted by the profession. Out of a doctrine, then, which we viewed with contempt for its ultraism, we are constrained to admit much good has arisen. In addition to and from these causes men have been led to think and reflect — atmospheric and terrestrial causes of disease have been carefully investigated, and the curative influence of remedies in new combinations patiently studied — theory has yielded to the known truths of observation, and our hospitals are the great storehouses of facts from which floods of light are pouring out to bless and preserve mankind—science is rapidly drawing aside the veil which has obscured our mental vision, and the light of truth and knowledge is opening a new era.

It may not be improper to mention yet another—the extraordinary excitement of the nervous system in this the most extraordinary of all ages, when the millions of our busy population are swaying to and fro under the mightiest impulses that ever controled the human mind or ruled its actions—when men rush to the sea, and upon its surface in multitudes for the rich returns and gains of a prosperous commerce—when the great prairies of our newly-opened territories are dotted with human beings seeking new homes—when machinery has attained the highest perfection, and when the human mind is wrought to its highest tension in every pursuit that enriches, enlightens or advances society. Is it wonderful that in such an excited state, the opiate and tonic treatment of disease is forced upon the minds of medical men?

"Already commences a new order of famous ages."
"*Magnus ab integro sæculorum nascitur ordo.*"

It was formerly rarely the case that any disease was treated many days without a salivation. This practice very generally obtained and was resorted to in acute as well as chronic diseases. It arose from an opinion advanced I think by John Hunter, that no two diseases could exist at the same time in the same system, and that when it was brought under the influence of mercury the disease would be displaced; hence it became the object of the physician, in every disease considered of a grave character, to ptyalise as speedily as possible. Tumid faces and the mercurial odor was to be found in almost every sick room. I have seen some frightful ravages of the soft and osseous tissues from the effects of mercury. Although patients rarely died from mercurialization, yet, when it was severe, the system was very much shattered, and a perfect state of health was rarely expected afterward. I have seen two or three children die from it, the whole lip and a portion of the cheek having been carried away. A few years ago I removed a portion of the inferior maxilla, which was necrosed, together with the teeth, from a little boy, six years of age, which was induced by ptyalism.

The remedy was very frequently of a more serious character than the disease; such being the case, it became a matter of no small importance to study those remedies which would soonest arrest it, or would most surely check the pain. A great variety of topical applications were in use, and internal remedies were constantly resorted to.

It was regarded a matter of so much importance, that I wrote my graduating thesis, *De usu emeticam in ptyalismo Cohibiendo*, in the use of emetics in arresting salivation. The indiscriminate use of mercury lessened to a certain extent public confidence in physicians, and ignorant quacks availing themselves of the prejudice, raised a wonderful hue and cry against all regular practitioners. Every published quack remedy was positively declared to be entirely free from all mercurial preparations.

It was to combat mercurial preparations that Thomsonianism, in part, had its origin. Prejudice carried a certain portion of community so far, that they were willing to adopt any apparently rational expedient, rather than submit to the chances of being salivated.

This Dr. Thomson had for a partner, a very shrewd quaker, named Horton Howard, who resided in Columbus, Ohio, and who had charge of the sale of all the family rights to practice medicine in the West. He became wealthy from it, and was a man of much influence. Before the cholera made its appearance in 1832, he promised the community in which he resided, that his lobelia and capsicum, with his hot baths, were omnipotent in this disease. It so happened, that the cholera, after it crossed the lakes from Canada in 1832, made its appearance in Columbus among the first towns. True to his promises, he diligently applied the instruments of his art, but in spite of lobelia, capsicum, and steam, his patients, as those of other men, died. A son-in-law, one of the most popular of American poets, died of it, under his treatment—the poet's wife and their children. A son and another daughter of the quaker, his own wife, and finally himself—all died martyrs to steam practice. Being a family of so much note and at the head of Western steam, it created a good deal of trembling among his brethren, for he had issued his confidence bulletins to his followers everywhere, and they regarded him infallible. This was during the palmy days of steam, and when they supported a college at Washington, with teachers in every department.

It was a paralyzing blow to the system in Ohio, and led men to doubt the truth of their vaunting promises. Notwithstanding, it flourished for many years after that. It had a sickly growth; its great head was gone; and, I presume, at this time in intelligent communities, few will anywhere be found advocating a doctrine of so few merits.

There is scarcely anything connected with the practice of medicine, which has undergone a greater and more favorable change within the last few years, than the dietetic rules prescribed for the sick. It was formerly the case, that those who were so unfortunate as to be sick, were doomed, not only to bleeding and puking and purging, but almost to absolute starvation. They had not only to contend against the disease, but against the remedies to which they were subjected for its cure. The strength of the patient was diminished as rapidly as possible, and I have no doubt, many died from actual exhaustion, caused by depressing remedial agents, and the lack of food enough to support life. I have, for many years, been in the habit, not only of permitting some food to be taken, but prescribing it as one of the means to aid a restoration of health. I know of no disease which will not be benefited by the use of some food every day, and here let me enter my solemn protest against all the slops, tasteless, nauseous, uninviting, and unsupporting, which are usually ordered for the sick. A healthy stomach would revolt at their introduction, and I am sure a sick one could only tolerate them as medicine and not as food.

Whatever is ordered for the sick should be nutritious and palatable, then the less quantity will be required. I have seen tapioca and sago and rice forced upon little children until the very sight of these would cause them to vomit, and I do not hesitate to declare, that I would regard it fortunate for the sick, if these articles of diet were forever blotted out. Let the various preparations of animal diet take their place, and we have that which is more palatable and more nourishing.

The tastes of the sick are sometimes, not only very capricious, but very peculiar, setting at defiance all dietetic reasoning. I once had a patient, a delicate young man, who had been suffering for a good while from indigestion. In fact, he could eat nothing which would not in a short time be rejected. He lost flesh and was very low spirited. I blistered his stomach and regulated his bowels and kept him down to the starving point, according to the nicest and most approved method of Wilson. I visited him daily until I became discouraged with my want of success. All that I did seemed of no avail. On one occasion, after he had been kept to my entire satisfaction, upon toast and tea and cracker, and a moiety of soft boiled egg and a little wine whey and chicken soup, so delicate from a mere dipping in of the fowl, he asked me if I would permit him to have a little cabbage soup. "*Cabbage soup*," I responded; "*no sir.*"

I was all the time puzzled in my own mind to know what sort of a mixture this could be, but I resolved that anything composed of cabbage would not do, hence my refusal. Next day, he ventured to remark again that he could relish a little cabbage soup. I denied him again. The third day, he repeated his cabbage soup request, to which I reluctantly assented, as I did not wish to be a *particeps criminis* in recommending what all human reason, under like circumstances, would declare to be hurtful. Well, at my next visit, he informed me that he had had a good time with his cabbage soup, that it had laid well upon his stomach, and that he felt greatly better. What could I do but to permit its continuance. He did continue it, and recovered rapidly from the day he commenced its use. I never inquired what his cabbage soup was made of, and to this day, I am ignorant of its composition. This will admonish us of the fact, that the appetite is oftentimes the very best index of what the stomach will bear, and what the system requires. It seems to stand as a sentinel, to invite that which is beneficial, and to reject that which is hurtful. To a certain extent, under all circumstances, I think it is our duty to gratify the taste. Since I have given more attention to this subject, I think I have had better success in the treatment of diseases. The case above cited, is but one in a thousand, and I have no doubt each one of you has had analogous cases.

I know of nothing on this subject, more valuable than that from the pen of one of the most distinguished and most successful practitioners of medicine, Dr. Graves, and I shall not deem it misspent time to read his views.

"In a disease like fever, which lasts frequently for fourteen, twenty-one, or more days, the consideration of diet and nutriment, is a matter of importance, and I am persuaded that this is a point on which much error has prevailed. I am convinced that the starving system, has, in many instances, been carried to a dangerous excess, and that many persons have fallen victims to prolonged abstinence in fever. This was one of the errors which sprung from the doctrines of those who maintained that fever depended on general or topical inflammation. They supposed that fever arose from inflammation, and immediately concluded that, to treat it successfully, it was necessary to reduce the system by depletion and low diet, and to keep it at this point during the whole course of the disease. Hence the strict regimen—*diete absolue*—of the disciples of the physiological school, and of those who looked on inflammation as the essence of fever."

The more the symptoms appeared indicative of inflammatory action, the more rigorous was the abstinence enforced. If a patient's face was flushed, or his eyes suffused, no matter what the stage of the fever was, they said, "here is inflammation of the brain, and nourishment will exasperate it." If he had red or dry tongue and abdominal tenderness, they immediately inferred the existence of gastro-enteritis, and all kinds of food, even the lightest, was strictly forbidden. That this proceeds from false notions of the nature of fever is beyond doubt, and I pointed out this fact many years ago, long before the appearance of Piorry's work. Let us in the first place examine the results of protracted abstinence in the healthy state of the system. Take a healthy person and deprive him of food, and what is the consequence? First, hunger, which, after some time, goes away, and then returns again. After two or three days the sensation assumes a morbid character, and instead of being a simple feeling of want and a desire for food, it becomes a disordered craving, attended with a dragging pain in the stomach, burning thirst, and sometime afterward epigastric tenderness, fever and delirium. Here we have the supervention of gastric disease and inflammation of the brain as the results of protracted starvation.

Now, these are in themselves very singular facts, and well deserving of being held in memory. Read the accounts of those who perished from starvation after the wrecks of the Medusa and the Alceste, and you will be struck with the horrible consequences of protracted hunger. I can refer you to more recent dates: those who were in the Isthmus of Darien with Lieut. Strain, and those who were saved for many days from the ill-fated Central America to die of starvation. You will find that most of the unhappy sufferers were raging maniacs, and exhibited symptoms of violent cerebral irritation.

Now, in a patient laboring under the effects of fever and a protracted abstinence, whose sensibilities are blunted, and whose functions are deranged, it is not at all improbable that such a person—perhaps also suffering from delirium or stupor—will not call for food, though requiring it, and that, if you do not press it upon him, and give it as a medicine, symptoms like those which arise from starvation in the healthy subject may supervene, and you may have gastro-enteric inflammation or cerebral disease, as the consequence of protracted abstinence. You may think that it is unnecessary to give food, as the patient appears to have no appetite, and does not care for it. You might as well think of allowing the urine to accumulate in the bladder, because the patient feels no desire to pass it. You are called on to interfere when the sensibility is impaired, and the natural appetite is dormant, and you are not to permit your patient to encounter the horrible consequences of inanition, because he does not ask for nutriment. I never do so. After the third or fourth day of fever, I always prescribe mild nourishment, and this is steadily and perseveringly continued through the whole course of the disease.

He further adds, in a lecture to a medical class: I have endeavored to impress upon you the fact that there can be no doubt that persons have been occasionally starved to death in fever, and laid before you some remarkable facts connected with the influence of protracted abstinence on the general system, as well as on the brain and digestive tube. I also endeavored to show that long-continued denial or want of food generates symptoms bearing a very close resemblance to those which are observed in the worst forms of typhus: pain in the stomach, epigastric tenderness, thirst, vomiting, determination of blood to the brain, suffusion of the eyes, headache, sleeplessness, and, finally, furious delirium, are the symptoms of protracted abstinence; and to these we may add, tendency to putrefaction of the animal tissues, chiefly shown by the spontaneous occurrence of gangrene of the lungs. It has been shown by Guislain, physician to the hospital for the insane at Gaud, that, in many instances, gangrene of the lungs has occurred in insane patients who have obstinately refused to take food. Out of twelve patients who died of inanition, nine had gangrene of the lungs. You perceive, then, that starvation may give rise to symptoms of gastric disease, to symptoms of cerebral derangement, and to mortification of the pulmonary tissue. It is not, therefore, wrong to suppose that gastric, cerebral, and even pulmonary symptoms may supervene, analogous to those which result from actual starvation.

An attentive observation of the foregoing arguments has led me, in the treatment of lung fevers, to adopt the advice of a country physician of great shrewdness, who advised me never to let my patients die of starvation. If I have more success than others in the treatment of fever, I think it is owing, in a great degree, to the adoption of this advice.

Much has been written on medicine, and much taught in our schools, which is not only valueless, but is positively injurious, for the reason that the young rely upon them as guides, and consequently are misled. I have never yet seen a young graduate from an Eastern school who at first had any true conception of Western diseases, or the best method of treating them. Bier, the oculist, I think, it was who said he had put out a hat full of eyes in learning the best method of operating for cataract. If we had the confessions of the young physicians who follow the books and the teachers exclusively—and at first they are compelled to do it—I am persuaded we should have many, very many extinguished lives instead of eyes. It is our duty, as physicians, no matter where our lot upon earth may be cast, to aid, in every possible way we can, to advance a science the most noble of all others. Of all, medicine is pre-eminently and justly the first. As much as human life excels in value money and property, by so much does it excel the law which has simply for its object the adjustment of human rights. While I would not underrate any other, I would demand for our own justice. I am proud, too, that every age has thrown into our ranks intellects of the highest and most brilliant order. I am also proud that the portals to science are open to all. Industry and perseverance, and a laudable ambition, will secure the highest honors of ours or any other profession. To the junior members who are my auditors I would offer the highest encouragement. Let all your leisure time be given to reading, and writing, and studying. Keep a record o every case you attend; employ your evenings in writing them out. You will find it pleasant and profitable, and the time will soon steal upon you when your opinions will be sought after as pearls of great price. Above all things, gentlemen, let me exhort you to do your own thinking. Consult the opinions of eminent writers, but let it be rather to assist and direct than to control. After fortifying yourselves with all necessary facts, suffer no one then to think for you, but maintain your manhood in wielding the thought which Deity has given to you, in common with others. I am an admirer of genius and talent—I will pay it homage wherever found. At the same time, I would declare that you and I, and every patient investigator of truth, is just as capable of thinking—just as capable of arriving at correct conclusions as those who are wielding public opinion in the great cities of the world.

A few years ago a poor boy of Indiana was apprenticed to a trade. He had no means—no education. He tried at his trade for a support, and having a high and holy purpose before him, stayed not his efforts until, unaided and unfriended, he had a diploma from a medical school. Still he toiled on. He was a devoted student, and became a contributor to the journals, and one of the most extensive translators from German and French periodicals of the day. These brought him into notice, for they compared favorably with the best contributions of the times. He established, by his own efforts, a medical journal. He became a professor of a college. He is now one of the Vice-Presidents of the U. S. Medical Association, is an accomplished scholar, and is at this time a professor in the medical college of Chicago, and one of the editors, within a few days, of the medical journal of your State. He richly merits every honor which has been conferred upon him, and, if he lives, he will enjoy a still prouder and a still more enviable reputation. Coming from my own State, I mention him with pride. Having been promoted to honors in yours, it is proper, on this occasion, and in the State of which he is now a citizen, to give you the name of Prof. Wm. H. Byford, as the gentleman referred to. If, at any time, any one present, or any of your friends, should visit Chicago, I beg you will make his acquaintance. I cite this instance, because he is one of us, a living monument of what industry and perseverance will accomplish.

And now, gentlemen, in conclusion, let me encourage you in the continuance of good works; and it affords me a particular pleasure to say that, as a profession, I know of none whose members discharge more faithfully their duties—whose aims and purposes are more laudable, and who live and labor with so much self-sacrifice as those of our own. To do good—to soothe the anxious heart—to allay pain and stay disease, is the physician's duty. Everywhere he is on the same holy mission. He is on the battle-field and in the pest-house. He is the fearless, sleepless sentinel at every threshold, to uplift the shadowing pall of death from its trembling, prostrate inmates. Where the pestilence reigneth most, there he is. Where all hearts are palsied with fear, as the desolating plague sweeps over the doomed to death, there is he to inspire hope to the anxious, to soothe the sorrowing, and to console the dying. All may not be great, but all may be good, and capable of doing good. To the humblest of the profession there is a ripened harvest—a field of sympathy, and sorrow, and disease, which invite the soothing voice, the kind and gentle look, and the hopeful agent of which he is the minister. There are sorrowing hearts which welcome his coming with grateful, confiding hope, and tearful eyes of gladness for all his words of consolation.

